# Diagnosis of Vitreoretinal Aspergillosis with Transvitreal Retinochoroidal Biopsy

**DOI:** 10.1155/2018/8306163

**Published:** 2018-12-02

**Authors:** Krishi Peddada, Nida M. Khan, Jascha Rubin, Haykanush Zakaryan, Yaobin Liu, Nikolay Popnikolov, Roshun Sangani, Weiye Li

**Affiliations:** ^1^Department of Ophthalmology, Drexel University College of Medicine, Philadelphia, PA, USA; ^2^Rittenhouse Hematology/Oncology, Philadelphia, PA, USA; ^3^Department of Radiology, Drexel University College of Medicine, Philadelphia, PA, USA; ^4^Department of Pathology & Laboratory Medicine, Drexel University College of Medicine, Philadelphia, PA, USA

## Abstract

Diagnosing culture-proven endophthalmitis is complicated by the insufficient yield of intraocular samples and the variety of etiologies which mimic true endophthalmitis. In cases of impending vision loss where vitreous biopsy cannot provide a definitive diagnosis, transvitreal retinochoroidal biopsy can be an effective next step. Our case is a 48-year-old male with B-cell acute lymphoblastic leukemia that presented with counting fingers vision, redness, and tearing of the left eye. Exam showed cell and flare with hypopyon as well as dense vitritis. The patient underwent diagnostic pars plana vitrectomy and vitreous culture was negative at the time. Flow cytometry demonstrated no malignant cells. However, the patient's vision and mental status continued to clinically decline despite being started on intravitreal and systemic antibiotic and antifungal therapy. Neuroimaging revealed rim-enhancing brain lesions. Transvitreal retinochoroidal biopsy was performed in an elevated area of the retina. The biopsy helped rule out malignancy and showed acute-angle, septate, branching hyphae characteristic of* Aspergillus fumigatus*. Ultimately, the vitreous biopsy, cultures, and a biopsy from the left frontal lobe brain abscess all confirmed this diagnosis as well. Transvitreal retinochoroidal biopsy can play a role in the diagnosis of a case of posterior uveitis and can be particularly effective in diagnosing a fungal endophthalmitis.

## 1. Introduction

Sampling of vitreous fluid is still considered as the gold standard to diagnose endophthalmitis even though vitreous samples have limited diagnostic yield [1]. The Endophthalmitis Vitrectomy Study found that 69.3% of eyes with confirmed endophthalmitis had positive cultures [2] and a study looking specifically at fungal endophthalmitis found that 70% of vitreous biopsies were positive [3]. Further complicating this situation is the fact that many patients with uveitis masquerade syndromes are actually mistaken for having culture-negative endophthalmitis. Uveitis masquerade syndromes include intraocular leukemia and lymphoma, conditions which can be life-threatening if not identified early [4]. In cases of impending vision loss with negative vitreous biopsy results, it is important for physicians to have other tools to distinguish between a culture-negative endophthalmitis and an intraocular malignancy.

The transvitreal retinochoroidal biopsy, first described by JR Griffin in 1975, is a type of surgical excisional biopsy that was originally utilized for diagnosing tumors of the posterior segment [5]. One large study showed that transvitreal retinochoroidal biopsy was able to diagnose malignancy in 97.3% of cases [6]. Another study of 29 uveitis patients with suspected intraocular lymphoma demonstrated that retinochoroidal biopsy made a histological diagnosis in 17 patients and excluded malignancy when combined with clinical data in 9 patients [7]. Retinochoroidal biopsy also plays a role in diagnosing infectious uveitis. In particular, it is indicated in those cases with nonrevealing workup, inflammation localized to the retina, and cases with vision threatening disease unresponsive to therapy [8]. However, the additional information provided by this procedure must be balanced against its risks which include subretinal hemorrhage, vitreous hemorrhage, and retinal detachment [9].

## 2. Case Report

**Figure 1:**
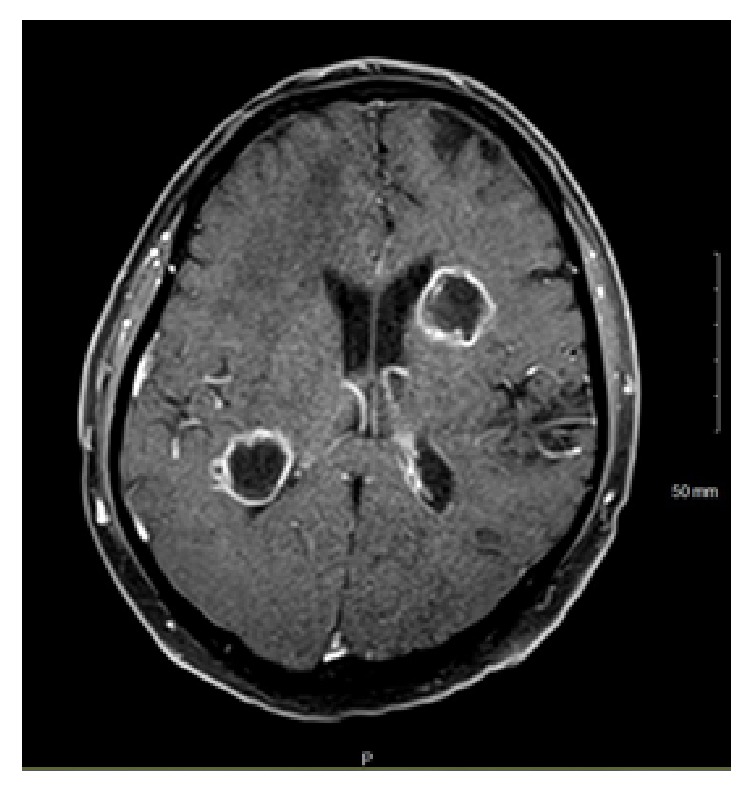
Axial contrast enhanced T1 WI brain MRI demonstrating rim enhancing lesions in the right peritrigonal white matter and left corona radiata.

**Figure 2:**
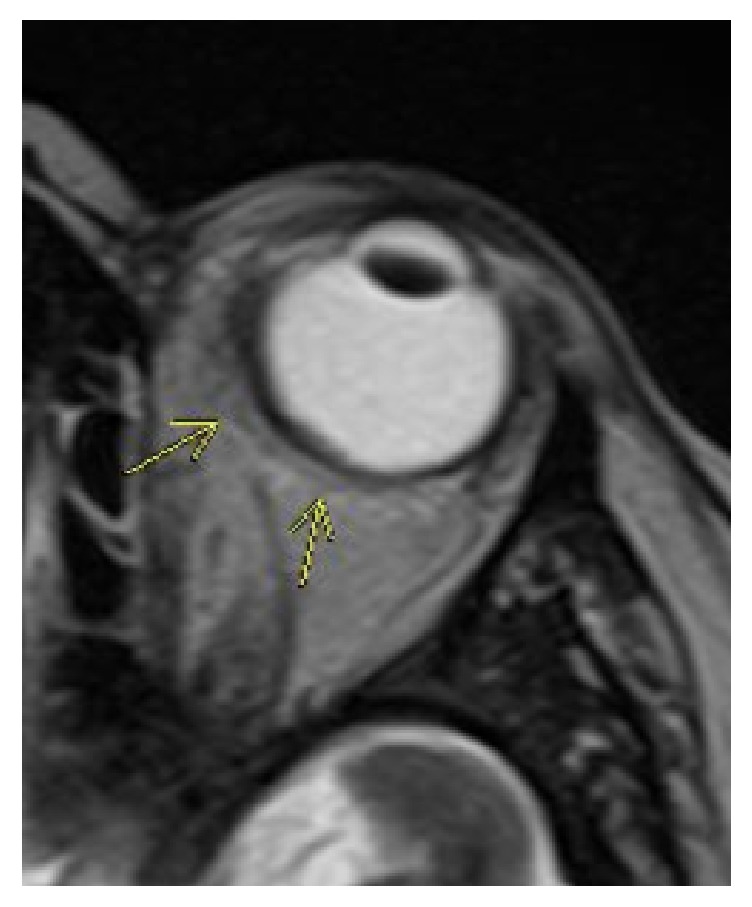
Axial T2 weighted image through the left orbit demonstrating irregular thickening of the posterior and medial wall of the left globe.

**Figure 3:**
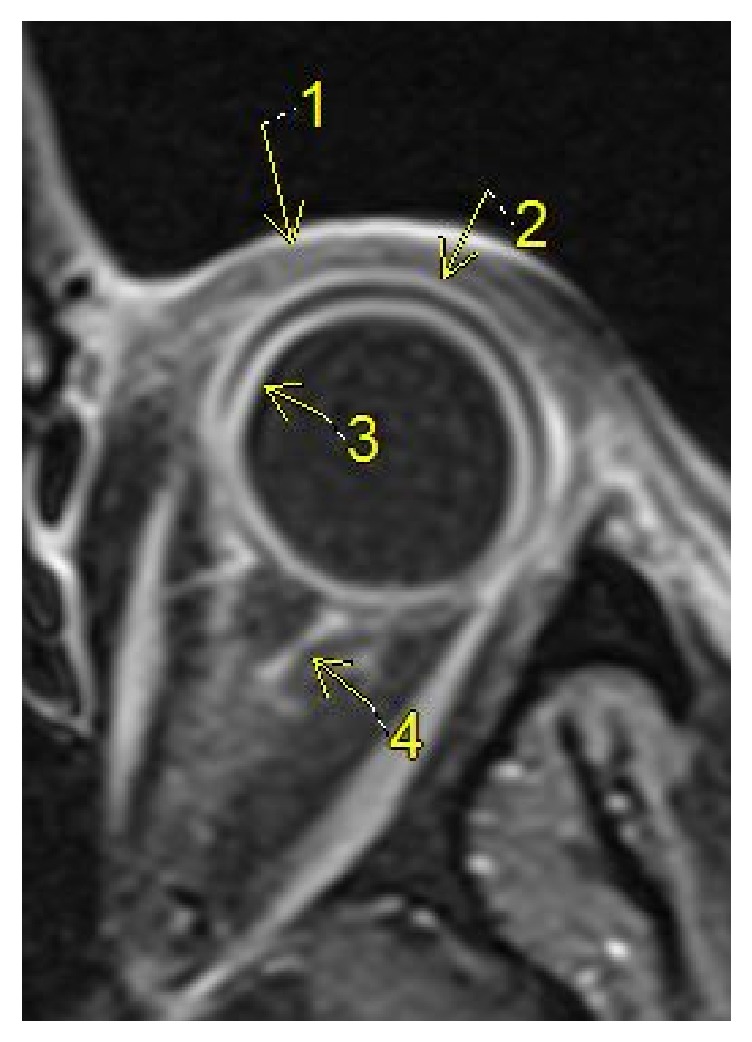
Axial contrast enhanced T1 WI through the left orbit demonstrating periorbital soft tissue swelling and enhancement (1) and smooth circumferential enhancement of sclera (2) and choroid (3); enhancement of the left optic nerve sheath and retrobulbar fat (4).

A 48-year-old male with B-cell acute lymphoblastic leukemia in partial remission receiving inpatient chemotherapy experienced left eye progressive vision loss, tearing, and redness for three days. On ophthalmological consultation, the left eye had a visual acuity of counting fingers at 1 foot with an afferent pupillary defect. Examination revealed 4+ cell and flare in the anterior chamber with 2mm hypopyon and dense vitreous haze in the posterior pole. Given the concern for endogenous endophthalmitis in an immunocompromised patient, a diagnostic pars plana vitrectomy was performed and a vitreous biopsy was obtained. Intravitreal antibiotics and antifungals were injected through the trocars. Intraoperatively, a pink nonpigmented 2 x 3 x 2mm (W x L x H) elevation was noted on the mid-peripheral superonasal retina. Given this lesion and the patient's declining mental status, imaging and cerebrospinal fluid sampling were performed. MRI brain illustrated rim-enhancing lesions with diffusion restriction in the right peritrigonal and left corona radiata (Figure 1) while orbital sections showed circumferential smooth enhancement in the left globe and optic nerve sheath (Figures 2 and 3). Cerebrospinal fluid removed from the right frontal ventricular reservoir through which the patient was receiving intrathecal chemotherapy showed no bacterial or fungal growth.

**Figure 4:**
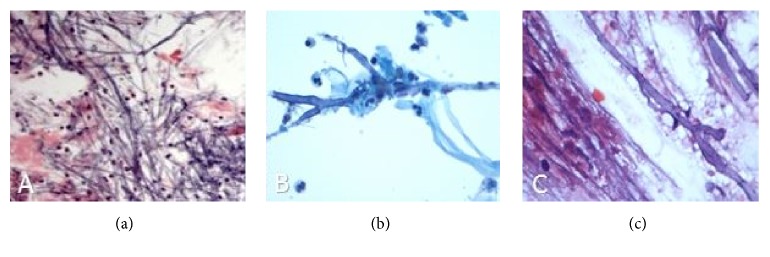
(a) PAS stain of the retinochoroidal biopsy demonstrates septate filamentous fungi with acute angle branching consistent with* Aspergillus fumigatus*. (b) Pap stain of the vitreous fluid shows acute inflammation, fibrin, and true septate hyphae with 45-degree branching. (c) Hematoxylin and eosin-stained squash preparation from the brain lesion demonstrates true hyphae with 45-degree branching in a background of necrotic debris.

Despite systemic use of antibiotics and antifungal medications, the patient's mental status over the next several days declined to the point where he could no longer follow commands. Repeat MRI brain 10 days later showed increasing sizes of the rim-enhancing lesions. Vision became “no light perception (NLP),” inferior retinal detachment was noted on exam, and the vitreous biopsy had still not grown any organisms. Both culture-negative endophthalmitis and uveitis masquerade syndromes such as intraocular malignancy still remained real life-threatening possibilities. Given the systemic risk of a bacteremia or malignancy, the ophthalmology team decided to proceed with a transvitreal retinochoroidal biopsy [6]. Septate filamentous fungi with acute angle branching characteristic of* Aspergillus fumigatus* were noted on retinochoroidal biopsy (Figure 4(a)). The patient was subsequently started on systemic voriconazole and amphotericin B. Two weeks later, culture from the initial vitrectomy demonstrated* Aspergillus fumigatus *(Figure 4(b)). Pathology from the brain tissue that was biopsied by neurosurgery demonstrated organisms morphologically consistent with* Aspergillus *as well (Figure 4(c)). Flow cytometry to rule out intraocular malignancy demonstrated an immunophenotypically normal cell population and cytology done on the vitreous biopsy did not show malignant cells. The patient was ultimately discharged from the hospital to a rehabilitation facility where he was informed that he would likely need enucleation of the left eye as an outpatient.

## 3. Discussion

Biopsy of chorioretinal tissue has historically been reserved for intraocular tumors that could not be diagnosed based on clinical features or noninvasive diagnostic techniques [5]. This was because retinochoroidal biopsy carried significant risks of choroidal bleeding, vitreous loss, and retinal detachment [10]. As new techniques have emerged to make retinochoroidal biopsy safer, it has become more accepted in the literature for indications such as posterior uveitis with nonrevealing workup, retinochoroidal inflammation, and sight-threatening disease [8]. However, there remain very few published articles that discuss retinochoroidal biopsy for posterior uveitis, likely reflecting physicians' discomfort in using the technique for this indication [11].

This case was appropriate for retinochoroidal biopsy for several reasons. The patient's deteriorating vision, inconclusive vitreous biopsy results at the time, poor response to antibiotics and antifungals, and poor overall health created a sense of urgency in establishing a diagnosis. Retinochoroidal biopsy was an effective modality because it could distinguish between the life-threatening diagnoses of culture-negative endophthalmitis and intraocular malignancy. Due to the acute nature of events, enucleation was not preferred. Had the patient had an intractable panophthalmitis with corneal opacity, scleral abscess, and ruptured globe, enucleation or evisceration may have been the only choice [12]. Ultimately, PAS stain of the retinochoroidal biopsy helped demonstrate the morphological features of acute, angle branching fungi with septate hyphae. Several tissues were ultimately biopsied including the frontal lobe of the brain, the vitreous, and the retina. Both Pap stain of the vitreous as well as hematoxylin and eosin stain of the brain tissue supported the diagnosis of* Aspergillus* as well.

Because of the inflammatory infiltrate seen in the vitrectomy specimen and the patient's history of leukemia, it was equally important to rule out primary intraocular lymphoma. Patients with primary intraocular lymphoma typically show features such as medium to large cell size, increased cellularity, nuclear apoptosis, and necrosis on cytology [13]. CD20 immunohistochemistry of cell blocks or destained cytospins is usually positive and flow cytometry demonstrates an immunophenotypically abnormal cell population [14]. Cytology of the vitreous and flow cytometry of the inflammatory infiltrates were performed in this case and did not show features characteristic for primary intraocular lymphoma.

CNS aspergillosis is a diagnosis that requires clinical suspicion, evidence on neuroimaging, and ultimately a histopathological specimen from the brain. Blood vessel invasion and thrombosis in the brain result in abscess formation such as that seen in our patient. Invasion of the retinal and choroidal vessels can create the type of retinal elevation that was observed in our patient superonasally [15].* Aspergillus* infections are also frequently associated with hematological malignancies [16]. Our patient also had many of the studied risk factors to develop* Aspergillus* endophthalmitis such as immunocompromise, neutropenia, lymphocytopenia, and malignancy [17].* Aspergillus* is virulent enough to afflict immunocompetent hosts and literature shows that the response rate to antifungal therapies such as voriconazole may be as low as 35% [18–20].

In conclusion, our patient presented with an aggressive, virulent* Aspergillus *endophthalmitis that led to total vision loss in a matter of days. His disease process was resistant to empiric intravitreal antifungal treatment. In this case, transvitreal retinochoroidal biopsy was critical in confirming infection and helping isolate the specific pathogen involved.
